# Supplementary material for: Heat Shock Protein DnaJ in Pseudomonas aeruginosa Affects Biofilm Formation via Pyocyanin Production
Source: Microorganisms. 2020 Mar 12;8(3):395. doi: 10.3390/microorganisms8030395 (PMC7143733; doi:10.3390/microorganisms8030395)
Supplement: Supplementary file 1 [file microorganisms-08-00395-s001.pdf]

microorganisms MDPI

Heat Shock Protein DnaJ in *Pseudomonas aeruginosa* Affects Biofilm Formation via Pyocyanin Production

Bo Zeng, Chong Wang, Pansong Zhang, Zisheng Guo, Lin Chen and Kangmin Duan

Table S1. Bacterial strains and plasmids used in this study.

**Stains**	**Relevant characteristic** [1]	**Source**
Pseudomonas aeruginosa PAO1		[1]
DH10B	F^- *mcrA* Δ(*mrr-hsdRMS-mcrBC*) φ80*lacZ*ΔM15 Δ*lacX74* *recA1 endA1 araD139* Δ(*ara-leu*)7697 *galU galK* λ– *rpsL*(Str^R) *nupG*	Invitrogen
danJ-M	PA4760 transposon mutant of PAO1; Gm^R	This study
**Plasmids**		
pMS402	Expression reporter plasmid carrying the promoterless *luxCDABE* gene; Kn^R ,Tmp^R	[2]
pAK1900	*E. coli-P. aeruginosa* shuttle cloning vector carrying plac upstream of MCS; Amp^R ,Cb^R	[3]
pKD-*phzM*	pMS402 containing *phzM* promoter region; Kn^R , Tmp^R	[4]
pKD-*phzS*	pMS402 containing *phzS* promoter region; Kn^R , Tmp^R	[4]
pKD-*phzA1*	pMS402 containing *phzA1* promoter region; Kn^R , Tmp^R	[4]
pKD-*phzA2*	pMS402 containing *phzA2* promoter region; Kn^R , Tmp^R	[4]
pKD-*pqsA*	pMS402 containing *pqsA* promoter region; Kn^R , Tmp^R	[5]
pKD-*lasI*	pMS402 containing *lasI* promoter region; Kn^R , Tmp^R	[6]
pKD-*lasR*	pMS402 containing *lasR* promoter region; Kn^R , Tmp^R	[6]
pKD-*rhlI*	pMS402 containing *rhlI* promoter region; Kn^R , Tmp^R	[6]
pKD-*rhlR*	pMS402 containing *rhlR* promoter region; Kn^R , Tmp^R	[6]
pKD-*flhF*	pMS402 containing *flhF* promoter region; Kn^R , Tmp^R	This study
pKD-*flhA*	pMS402 containing *flhA* promoter region; Kn^R , Tmp^R	This study
pAK-*dnaJ*	pAK1900 with a 1.1 kb fragment of *dnaJ* between XbaI and HindIII; Amp^R	This study

[1] Tmp^R, trimethoprim resistance; Gm^R, gentamicin resistance; Kn^R, kanamycin resistance; Amp^R, ampicillin resistance; Cb^R, carbencillin resistance.

Table S2. Primers used in this study.

Primer	Sequence (5′-3′) [1]	Restriction site
PA4760 up	AATAAGCTTGCATGATCCGCCACGC	HindIII
PA4760 down	GGCTCTAGACGGTCTGCTGAACGGC	XbaI
ARB1	GGCCACGCGTCGACTACTACNNNNNNNNNNGATAT	
P7-1	CTAACAATTCGTTCAAGCCG	
ARB2	GGCCACGCGTCGACTAGTAC	
P7-2	GGATGCGTCTAAAAGCCTGC	
flhF up	GCACTCGAGGACAAGGCTCCGAGG	XhoI
flhF down	ACAGGATCCGCAGCTCCACTTCCAG	BamHI
flhA up	CGGTCTCGAGTAGTTGGTGATTGGCG	XhoI
flhA down	AATGGATCCGACCACGTAGTTGCCG	BamHI

[1] Restriction sites are underlined.

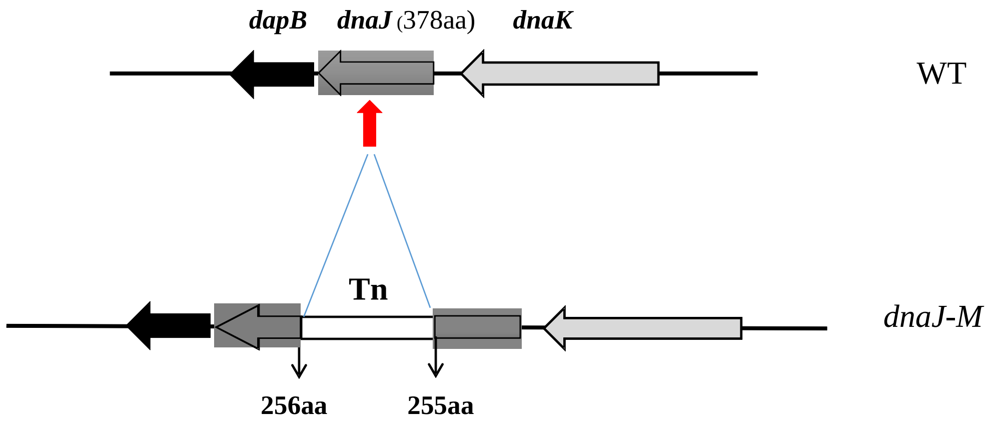

Figure S1. Schematic depiction of the genomic organization of *dnaJ-M*. Top: intact *danJ* gene on the chromosome in the wild type (WT). Bottom: *danJ* was disrupted by the transposon element (Tn). The insertion site is indicated with the DnaJ amino acid residues.

References

1. Stover, C.K.; Pham, X.Q.; Erwin, A.L.; Mizoguchi, S.D.; Warrener, P.; Hickey, M.J.; Brinkman, F.S.; Hufnagle, W.O.; Kowalik, D.J.; Lagrou, M., et al. Complete genome sequence of Pseudomonas aeruginosa PA01, an opportunistic pathogen. Nature 2000, 406, 959-964.
2. Duan, K.; Dammel, C.; Stein, J.; Rabin, H.; Surette, M.G. Modulation of Pseudomonas aeruginosa gene expression by host microflora through interspecies communication. Mol Microbiol 2003, 50, 1477-1491.
3. Poole, K.; Neshat, S.; Krebes, K.; Heinrichs, D.E. Cloning and nucleotide sequence analysis of the ferripyoverdine receptor gene fpvA of Pseudomonas aeruginosa. J Bacteriol 1993, 175, 4597-4604.
4. Yan, R.; Hu, S.; Ma, N.; Song, P.; Liang, Q.; Zhang, H.; Li, Y.; Shen, L.; Duan, K.; Chen, L. Regulatory Effect of DNA Topoisomerase I on T3SS Activity, Antibiotic Susceptibility and Quorum-Sensing-Independent Pyocyanin Synthesis in Pseudomonas aeruginosa. Int J Mol Sci 2019, 20, 1116, doi:10.3390/ijms20051116.
5. Liang, H.; Li, L.; Kong, W.; Shen, L.; Duan, K. Identification of a novel regulator of the quorum-sensing systems in Pseudomonas aeruginosa. FEMS Microbiol Lett 2009, 293, 196-204.
6. Duan, K.; Surette, M.G. Environmental regulation of Pseudomonas aeruginosa PAO1 Las and Rhl quorum-sensing systems. J Bacteriol 2007, 189, 4827-4836, doi:10.1128/JB.00043-07.
